# Supplementary material for: Resolving the full spectrum of human genome variation using Linked-Reads
Source: Genome Res. 2019 Apr;29(4):635–45. doi: 10.1101/gr.234443.118 (PMC6442396; doi:10.1101/gr.234443.118)
Supplement: Supplemental Material [file supp_gr.234443.118_Supplemental_longranger-2.2.zip › longranger-2.2.tar.bz2!/lr2.2-public/lib/stan/cmdstan/stan/doc/api/html/bfgs__linesearch_8hpp.html]

Stan: src/stan/optimization/bfgs\_linesearch.hpp File Reference


|  |  |
| --- | --- |
|  | Stan  2.5.0  probability, sampling & optimization |


- Main Page
- Related Pages
- Namespaces
- Classes
- Files

- File List
- File Members

All Classes Namespaces Files Functions Variables Typedefs Enumerations Enumerator Friends Macros Pages

- src
- stan
- optimization

Namespaces |
Functions

bfgs\_linesearch.hpp File Reference

`#include <cmath>`  
`#include <cstdlib>`  
`#include <string>`  
`#include <limits>`  
`#include <boost/math/special_functions/fpclassify.hpp>`

Go to the source code of this file.

|  |  |
| --- | --- |
| Namespaces | |
|  | stan |
|  | Probability, optimization and sampling library. |
|  | |
|  | stan::optimization |
|  | |

|  |  |
| --- | --- |
| Functions | |
| template<typename Scalar > | |
| Scalar | stan::optimization::CubicInterp (const Scalar &df0, const Scalar &x1, const Scalar &f1, const Scalar &df1, const Scalar &loX, const Scalar &hiX) |
|  | Find the minima in an interval [loX, hiX] of a cubic function which interpolates the points, function values and gradients provided. More... |
|  | |
| template<typename Scalar > | |
| Scalar | stan::optimization::CubicInterp (const Scalar &x0, const Scalar &f0, const Scalar &df0, const Scalar &x1, const Scalar &f1, const Scalar &df1, const Scalar &loX, const Scalar &hiX) |
|  | Find the minima in an interval [loX, hiX] of a cubic function which interpolates the points, function values and gradients provided. More... |
|  | |
| template<typename FunctorType , typename Scalar , typename XType > | |
| int | stan::optimization::WolfeLineSearch (FunctorType &func, Scalar &alpha, XType &x1, Scalar &f1, XType &gradx1, const XType &p, const XType &x0, const Scalar &f0, const XType &gradx0, const Scalar &c1, const Scalar &c2, const Scalar &minAlpha) |
|  | Perform a line search which finds an approximate solution to:   satisfying the strong Wolfe conditions: 1)  2)  where  is the gradient of f(x). More... |
|  | |


---

[ Stan Home Page ]

*© 2011–2014,
Stan Development Team.*
